# A web-based system for creating, viewing, and editing precursor mass spectrometry ground truth data

**DOI:** 10.1186/s12859-020-03752-7

**Published:** 2020-09-23

**Authors:** Jessica Henning, Rob Smith

**Affiliations:** Department of Computer Science, University of Montana, 32 Campus Drive, Missoula, MT 59812 USA

**Keywords:** Mass spectrometry, Mass spectrometry viewer, MS1 feature finding, Open source mass spectrometry software

## Abstract

**Background:**

Mass spectrometry (MS) uses mass-to-charge ratios of measured particles to decode the identities and quantities of molecules in a sample. Interpretation of raw MS depends upon data processing algorithms that render it human-interpretable. Quantitative MS workflows are complex experimental chains and it is crucial to know the performance and bias of each data processing method as they impact accuracy, coverage, and statistical significance of the result. Creation of the ground truth necessary for quantitatively evaluating MS1-aware algorithms is difficult and tedious task, and better software for creating such datasets would facilitate more extensive evaluation and improvement of MS data processing algorithms.

**Results:**

We present JS-MS 2.0, a software suite that provides a dependency-free, browser-based, one click, cross-platform solution for creating MS1 ground truth. The software retains the first version’s capacity for loading, viewing, and navigating MS1 data in 2- and 3-D, and adds tools for capturing, editing, saving, and viewing isotopic envelope and extracted isotopic chromatogram features. The software can also be used to view and explore the results of feature finding algorithms.

**Conclusions:**

JS-MS 2.0 enables faster creation and inspection of MS1 ground truth data. It is publicly available with an MIT license at github.com/optimusmoose/jsms.

## Background

Mass spectrometry (MS) is a powerful tool for the analysis of molecular components (such as proteins, peptides, lipids, and metabolites) in biological samples across a broad range of applications [1]. MS experiments generate datasets consisting of millions of 3-D points consisting of mass-to-charge (*m/z*), retention time (RT), and intensity. MS experiments require the mapping of all or some of these points to signal groups that correspond to a single (or multiple, in the case of isomers) molecules at a given charge state.

This process, called feature detection, has been addressed by numerous algorithms, commercial software, and public software such as MaxQuant [2], MZMine 2 [3], CentWave (XCMS) [4], MatchedFilter (XCMS) [4], and Massifquant (XCMS) [5]. Though many chemical standards and qualitative QC evaluations have been conducted, feature-level quantitative evaluation requires feature-level ground truth-meaning raw data points manually curated into features, whether extracted ion chromatograms or isotopic envelopes. These types of evaluations have not been common [6], mostly due to the fact that this type of ground truth data is very difficult to generate.

Manual feature curation is a laborious and subjective task which requires software to make it as principled and fast as possible.

A system for producing MS1 ground truth annotations requires several functions:

- It must parse, load, store, and retrieve MS1 data.
- It must efficiently display many points on the screen.
- It must display points in representative subsets, as not all points can be rendered on the screen at once.
- It must output the data in easy-to-port formats.
- It must provide the user with efficient navigation of the data (zoom and shifting to the right, left, up, or down).

JS-MS [7] is one of very few MS data tools that can be easily installed on multiple operating systems without the need for excessive dependencies or onerous compilation (another, for example, is pyOpenMS [8]).

JS-MS is a browser-based JavaScript viewer and Java server designed to load and view MS1 data. It provides useful navigation tools such as the ability to zoom in, zoom out, pan up-down-left-right, and toggle between 2-D and 3-D views.

The server component communicates with the view through a simple JSON API, which makes it interchangeable with any other server that implements the same API. The server responds to queries for specific (*m/z*, RT) windows. Each query includes a requested limit on the number of points returned, which invokes the server’s algorithm for selecting a representative subset of points, allowing for the user to view the characteristics of the data while only seeing a portion of the points in the given (*m/z*, RT) region. The server implements the MzTree data structure [9], which is a modified R-Tree that organizes the MS1 points in alternating sorting of *m/z* and RT to provide fast query response whether the data region requested is primarily across *m/z*, RT, or both.

JS-MS is packaged as a single self-contained JAR, and the only dependencies are the Java Runtime Environment (JRE) and a web browser, both typically already available on any computer.

Since the publication of JS-MS, our group has substantially extended the software. In addition to loading, viewing, and navigating MS1 data in 2-D and 3-D, JS-MS 2.0 extends JS-MS by providing tools for creating, editing, and viewing annotations of extracted ion chromatograms (called isotopic traces hereafter) and features (called isotopic envelopes hereafter). These tools facilitate inspection and modification of algorithms for isotope trace and isotopic envelope annotation, as well as the creation of manually annotated MS1 ground truth.

To date, our group has used JS-MS 2.0 to create the first ever quantitative ground truth dataset for MS1 data [10], as well as the first quantitative evaluation of algorithms that group traces into isotopic envelopes [11]. We are now releasing JS-MS 2.0 in hopes that others will use it to generate more ground truth to enable new and more extensive quantitative evaluations of MS1 algorithms.

**Fig. 1:**
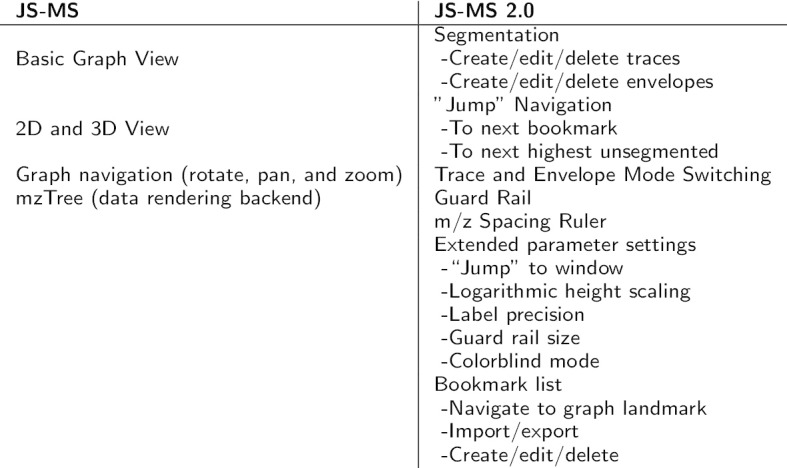
JS-MS 2.0 includes extensive additional features that facilitate faster and more accurate inspection and modification of feature-level point associations in MS1 data

The features added for JS-MS 2.0 make it useful for tasks extending beyond manual ground truth creation (see Fig. 1). For example, it allows mining, visualizing, and dissecting complex MS data for lists of targets represented as *m/z*, RT coordinates. This is vital for tasks that require visual inspection of isotopic envelopes, such as when checking the quality of putative biomarker quantification, when evaluating co-isolation levels on features of interest, when monitoring chromatography performance, and when evaluating MS data acquired on known targets.

## Implementation

JS-MS 2.0 extends the original JS-MS implementation through many extensions to the view, additions to the MzTree data storage and retrieval system, and additional API calls to the server.

The JS-MS 2.0 view provides extensions that enable annotations to be displayed, recorded, and edited, as well as helper tools that facilitate fast annotation decisions and annotation inspection.

The original application included logic that colorized signals based on intensity. In JS-MS 2.0, additional logic defines color based on trace or envelope membership (in each mode, respectively) such that proximate signals have different colors.

JS-MS 2.0 has a new ruler feature written in Three.js [12] that calculates the expected *m/z* intervals of an envelope of a given charge state. Vertical lines are drawn where each trace should appear with *m/z* gaps adjusting according to the charge state indicated by the user. Bounds checking prevents the ruler from extending beyond the plot range. Ratios are calculated to enable scaling of the ruler on zoom. On-click events activate the ruler when any number key is pressed and deviated when the tilde key is pressed.

The bookmark list is a new feature implemented in JavaScript and HTML that provides a means of storing, editing and applying (*m/z*, RT) coordinates to facilitate fast navigation to regions of interest. JavaScript calls dynamically add and remove rows from the table, edit entries in the table, and store the table in the cache. A parser function inputs a tab separated text file of bookmarks. An export function writes out the current bookmark list in the same tab separated format.

Annotations of isotopic traces use a rectangle tool that is written in Three.js. On-click and draw JavaScript functions map the region of data traced by the user’s mouse to (*m/z*, RT) coordinates used to update the point membership. An on-click event for the control key is used to toggle the function of the rectangle to remove points from a trace.

Annotations of isotopic envelopes require the selection of one or more traces to group into an envelope. Because users will not likely click directly on a point in a trace, trace selection relies on a JavaScript function that finds the closest trace within a threshold to the point clicked. Alternatively, users can click and drag a line through multiple traces to perform the same process across a set of points. An on-click event for the control key is used to toggle the behavior of the on-click event to remove one or more traces from an envelope.

Since traces tend to occur in straight lines along a given *m/z*, guide lines can be drawn using the Guard Rails feature. Using Three.js parallel lines are drawn along the *m/z* for a given *m/z* width with the appropriate projection in 2-D or 3-D mode, allowing the feature to persist independent of graph panning, rotation, or zoom. On-click events activate the tool with the ‘g’ key and deactivate with the ‘h’ key.

JS-MS 2.0 also includes extra controls for the user to modify view parameters such as point threshold, logarithmic height scaling, and label precision. The point threshold is a function implemented in Java that limits the number of points rendered in a given view, selecting a representative subset of points using the weighted striding algorithm [9]. Applying a point threshold allows for faster load time and graph navigation. Logarithmic height scaling is implemented with JavaScript and mathematical functions that scale point intensity by a logarithmic factor to facilitate greater contrast between signal and background noise. Label precision is also implemented with JavaScript to decrease or increase the level of precision for (*m/z*, RT) coordinates. This function rounds the (*m/z*, RT) coordinates to the desired accuracy from the user.

The MzTree data structure is a modified R-Tree [9] that interleaves data partitions sorted by RT and *m/z* for fast queries in either dimension. The previously published version of the data structure did not include the fields required for annotation (such as isotope trace ID and isotopic envelope ID). The previous version also lacked a new index of points sorted by intensity which is used in the jump button (discussed later).

The original JS-MS featured an HTTP API that included functions to retrieve a subset of points given an (*m/z*, RT) window, with an optional limit on the number of points returned. The API was extended to include trace and envelope annotation fields in the returned JSON data as well as functions to assign and edit those fields.

## Results

The user interacts with JS-MS through three main interfaces: the graph interface, the control panel, and the parameter panel.

### Graph interface

The principle purpose of the graph interface is twofold: First, it displays mass spectrometry points and isotopic trace and isotopic envelope annotations of these points. Second, it provides the controls for recording and editing these annotations.

Users have the ability to navigate to areas of interest through several means. First, the user can pan, zoom in and out, and toggle between 2-D and 3-D views of areas of their choice.

**Fig. 2:**
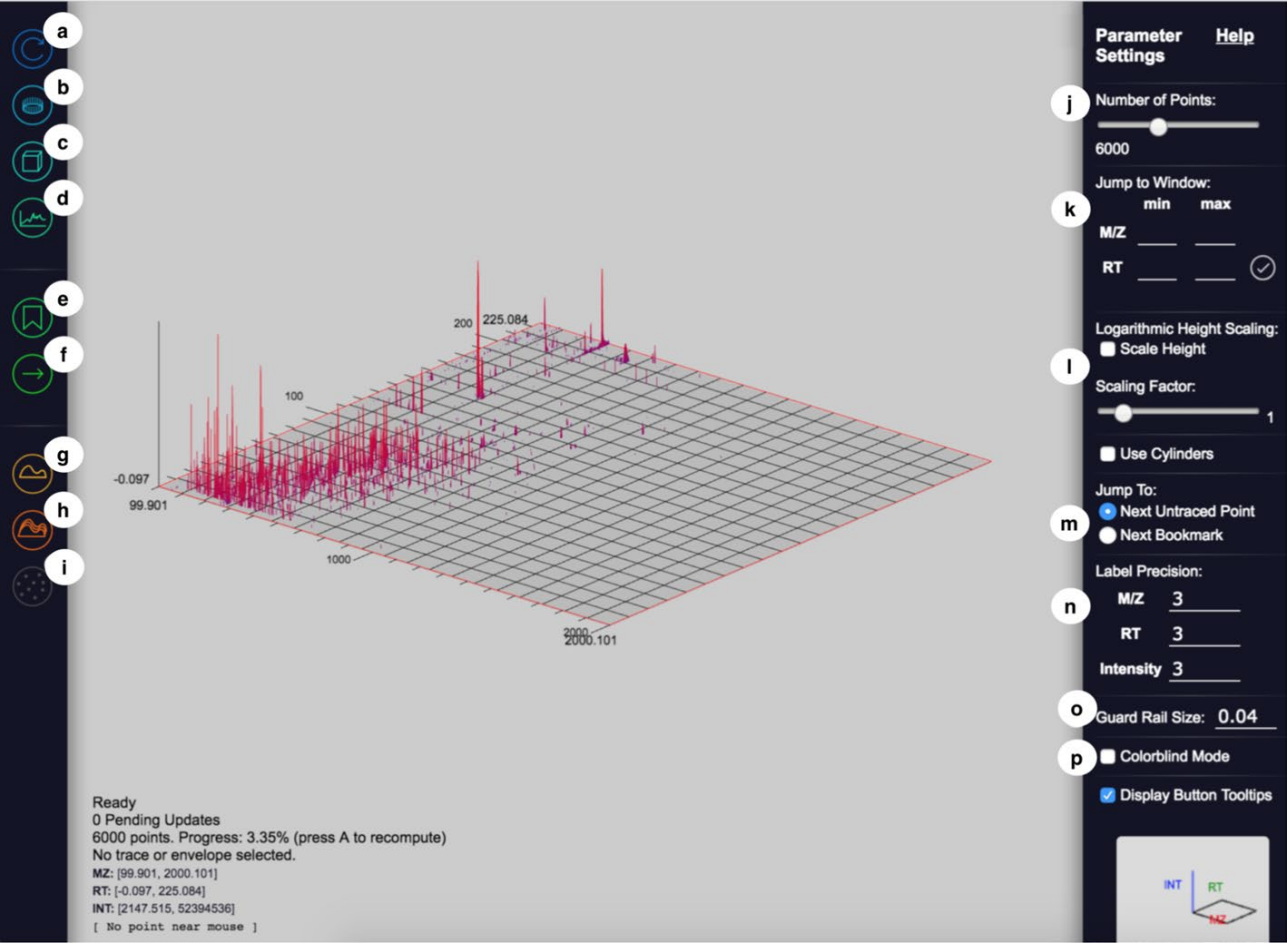
JS-MS 2.0 Bookmark List allows users to easily create, navigate to, import, and export a list of (*m/z*, RT) coordinates

Second, users can build a bookmark list to enumerate data points of interest by listing (*m/z*, RT) coordinates or using the “select current location” button (see Fig. 2e), which will add the current location to a list of one-click navigable data regions called the jump list. The jump list provides a useful mechanism for rapidly navigating to areas of interest. For example, if third party software provides a list of regions with poor feature detection, low intensity features, or known compounds, the jump list can be used to quickly iterate through the inspection of each corresponding data region. The bookmark interface features a button for importing and exporting bookmark lists in .tsv format (see Fig. 2a, b), and each bookmark entry can be edited or deleted (see Fig. 2c, d).

Third, users can use the jump button to navigate to other data areas. There are two functions associated with the jump button that can be toggled in the parameter panel. The first jump function is to jump to the next highest intensity point that is not part of an annotation. By clicking it, the graph will respond by displaying the area around the point, which will be denoted by an X on the graph. The second jump function is used to enumerate through the graphs of the areas around the points listed in the bookmark list. Using this feature, users can quickly inspect many envelopes or other data features in which they may be interested.

Fourth, a convenient “jump to window” mechanism on the control panel allows users to specify the exact window that they would like to display. This functionality facilitates the creation of reproducible graphs for inspection and publication.

**Fig. 3:**
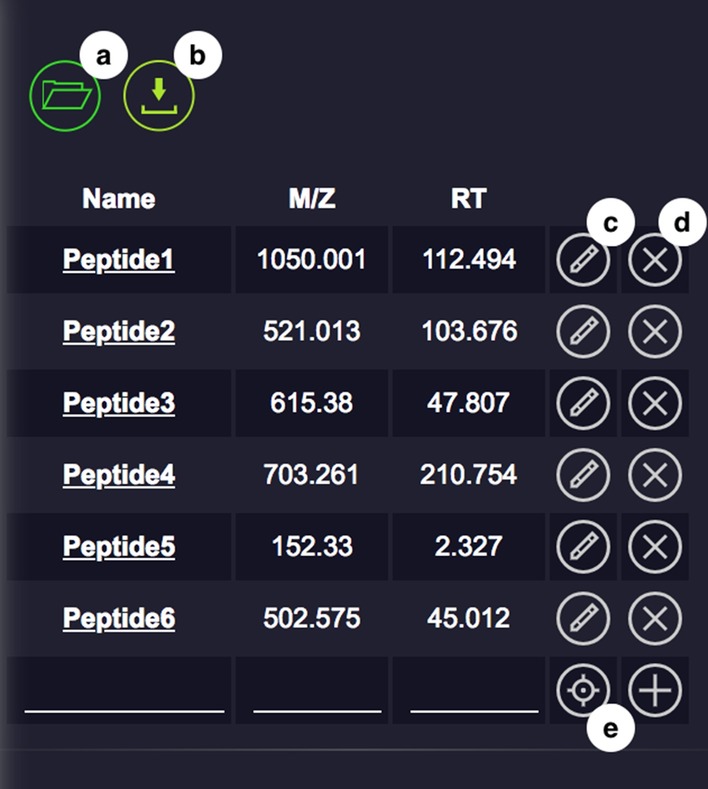
JS-MS 2.0 interface includes a control panel (left), graph interface (center), and parameter panel (right)

### Control panel

The control panel contains interfaces for users to define the graph’s behavior to match their intended purpose.

- Refresh (see Fig. 3a). This button reloads the data on the view from the server.
- View all data (see Fig. 3b). This button displays the entire data set.
- Toggle 2-D/3-D (see Fig. 3c). This button switches the graph display mode and redraws the graph.
- Ion current view (see Fig. 3d). This button rotates the view to a 2-D projection of the 3-D view such that the x-axis is *m/z* and the y-axis is intensity.
- Bookmarks (see Fig. 3e). This button shows or hides the bookmark interface.
- Jump (see Fig. 3f). This button’s functions are described above in “View.”
- Trace mode (see Fig. 3g). This button activates annotation mode, which is described below.
- Envelope mode (see Fig. 3h). This button activates isotopic envelope annotation mode, which is described below.
- Mark as noise button (see Fig. 3i). This button is used to indicate that all distinguishable signals in a view have been annotated and is further described below.

### Parameter panel

The parameter panel contains settings that adjust the view.

**Fig. 4:**
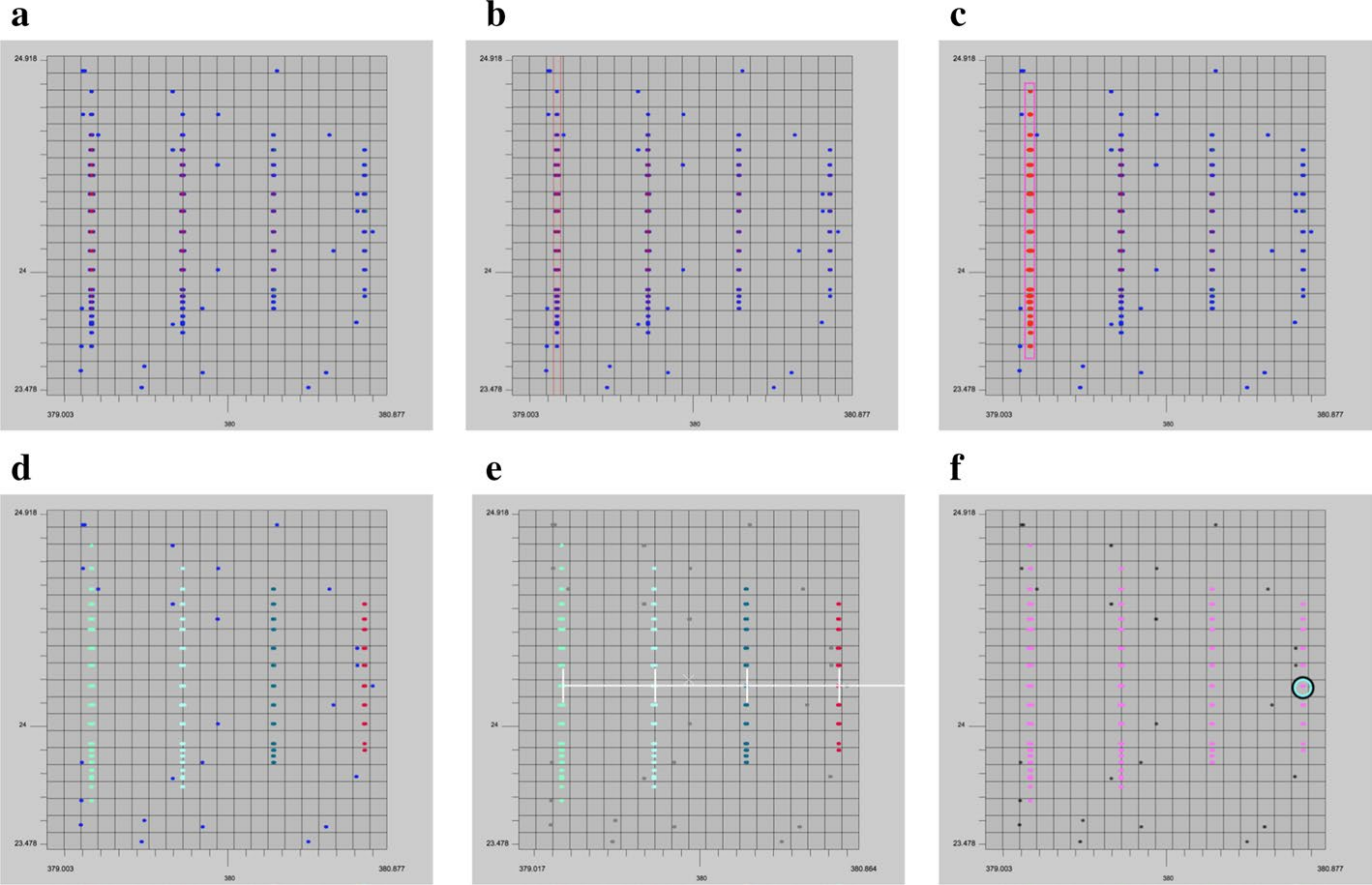
The annotation process for isotopic traces and envelopes. **a** Signals are shown in color based on intensity. **b** Guard rails are used to help distinguish which signals belong in an isotopic trace. **c**, **d** In trace mode, users mark which signals belong to an isotopic trace. **e** The ruler shows a user specified charge state to measure the isotopic traces within an isotopic envelope. **f** In envelope mode, users can specify all the traces belonging to an envelope

- Point threshold (see Fig. 3j). Users can control how many points are rendered for the given view. In the event the setting is lower than the actual number of points, JS-MS selects a representative subset of points using the weighted striding algorithm described in [9].
- Set view window (see Fig. 3k). This tool allows users to obtain a consistent view given the same specified (*m/z*, RT) window.
- Height scaling (see Fig. 3l). The height scaling slider changes the intensity and colorization scaling of points in order to more effectively display low intensity points in 3-D mode.
- Jump options (see Fig. 3m). This button specifies which jump function is active.
- Label precision (see Fig. 3n). This setting controls how many digits of precision are displayed on the graph.
- Guard rail size (button see Fig. 3o, use case see Fig. 4b). The guard rail is a set of parallel lines that can be displayed for a given *m/z* to assist in annotating an isotopic trace. This setting controls the width of the lines.
- Colorblind mode (see Fig. 3p). The colors used by the system can be limited to those visible to colorblind people.

### Annotation

#### Isotopic trace mode

When a user enters isotopic trace mode, they are given the option to create a new trace or select an existing trace to edit. Each time a new trace is created, the trace is given an ID and color. Users select the points belonging to the trace by clicking and dragging a rectangle over the desired points to highlight them in the given color (see Fig. 4c). The same procedure is used to edit an existing trace, only the control key is depressed while drawing the rectangle.

#### Isotopic envelope mode

After the user has identified isotopic traces, they can group them together with isotopic envelope mode. Similar to isotopic trace mode, this mode creates a new envelope ID and color for each new envelope created. The user then selects all isotopic traces that belong to the same group (see Fig. 4f). Isotopic traces can be grouped by clicking each trace or simply by dragging a line across all traces in an envelope. To help the user distinguish which isotopic traces belong together, the ruler tool shows *m/z* intervals corresponding to specific charge states. The ruler will appear wherever the mouse is placed when users select a number from the keypad. The ruler moves with the graph as the user zooms or pans, and will remain present until the user hits the tilde key. The *m/z* distance displayed is 1/z, where z is the number selected and the charge state of a hypothetical compound at the given mass (see Fig. 4e). Users can also toggle between 2-D and 3-D mode while in either isotopic trace or isotopic envelope mode to ensure peak alignment. Isotopic traces can be added to existing isotopic envelopes at any time following this procedure and they can be removed in the same way while depressing the control key.

#### Mark as noise button

When all distinguishable points in the current view have been annotated the user can mark all other points in the view as noise. When a point is marked as noise it will be colored gray in the view and given an ID of − 1 when exported to .csv. To prevent users from marking unseen points as noise, the graph view must be displaying a number of points below the point threshold to ensure that the user is viewing every point within the (*m/z*, RT) coordinates and none are hidden.

## Discussion

Algorithm performance can significantly affect the results of mass spectrometry experiments [13], and as such, a performance evaluation should be part of any new algorithm publication. Existing algorithm evaluations typically report performance based on consensus results [14]. While consensus results provide a qualitative gauge of how similar result sets are, they do not answer the critical question-how accurate are these results? Instead, consensus results measure how closely new algorithms perform compared to prior ones. While a positive consensus result does measure similarity to previous performance, it can’t distinguish whether differences are due to improvement or decline in accuracy.

The creation of benchmark datasets for MS1-aware mass spectrometry algorithms with JS-MS 2.0 will enable a new workflow for MS1 MS algorithm evaluation that includes quantitative evaluation. New algorithms can be designed using information derived from ground truth annotations created with JS-MS. Once implemented, their performance can be evaluated in terms of, for instance, *m/z* accuracy of traces annotated, to demonstrate clear improvement over existing algorithms. These evaluations will demonstrate strengths and weaknesses to reviewers and users alike.

One such benchmark dataset is currently being constructed by our group for isotopic trace algorithms, and the community is invited to use JS-MS 2.0 to create many more such datasets for any and all MS1-aware applications (such as quantification, centroiding, etc.).

## Conclusion

JS-MS 2.0 provides a dependency-free, browser-based, cross-platform solution for creating MS1 ground truth. Novel interfaces allows users to quickly add, edit, import, and export isotope trace and isotopic envelop annotations.

While other MS viewers do not allow users to easily navigate MS datasets, the innovative navigation tools in JS-MS 2.0 give users the ability to inspect and annotate any area of signals quickly with pan, zoom, and bookmark lists. It combines interactive 2-D and 3-D plots with fast, easy to use navigation tools allowing for manual annotation of even the largest MS datasets. The creation of ground truth MS datasets will benefit algorithm development, quantitative evaluation, and help practitioners assess strengths and weaknesses of existing workflows.

JS-MS 2.0 is implemented as a JavaScript frontend and Java backend, it is lightweight with browser-based cross-platform compatibility.

## Availability and requirements

*Project name*: JS-MS 2.0.

*Project home page*:

http://www.github.com/optimusmoose/jsms.

*Operating system(s)*: Platform independent.

*Programming language*: Java, JavaScript.

*Other requirements*: An internet browser.

*License*: MIT.

*Any restrictions to use by non-academics*: None.

## Data Availability

JS-MS is publicly available with an MIT license at github.com/optimusmoose/jsms.

## Abbreviations

MS: Mass spectrometry
RT: Retention time
*m/z*: Mass-to-charge ratio

## References

[1] Cole RB (1997). Electrospray ionization mass spectrometry: fundamentals, instrumentation, and applications.

[2] Cox J, Mann M (2008). MaxQuant enables high peptide identification rates, individualized ppb-range mass accuracies and proteome-wide protein quantification. Nat Biotechnol.

[3] Pluskal T, Castillo S, Villar-Briones A, Orešič M (2010). MZmine 2: modular framework for processing, visualizing, and analyzing mass spectrometry-based molecular profile data. BMC Bioinform.

[4] Tautenhahn R, Bottcher C, Neumann S (2008). Highly sensitive feature detection for high resolution LC/MS. BMC Bioinform..

[5] Conley CJ, Smith R, Torgrip RJ, Taylor RM, Tautenhahn R, Prince JT (2014). Massifquant: open-source Kalman filter based XC-MS isotope trace feature detection. Bioinformatics.

[6] Smith R, Ventura D, Prince JT (2013). Novel algorithms and the benefits of comparative validation. Bioinformatics.

[7] Rosen J, Handy K, Gillan A, Smith R (2017). JS-MS: a cross-platform, modular Javascript viewer for mass spectrometry signals. BMC Bioinform..

[8] Röst HL, Schmitt U, Aebersold R, Malmström L (2014). Pyopenms: a python-based interface to the OpenMS mass-spectrometry algorithm library. Proteomics.

[9] Handy K, Rosen J, Gillan A, Smith R (2017). Fast, axis-agnostic, dynamically summarized storage and retrieval for mass spectrometry data. PLoS ONE.

[10] Henning J, Tostengard A, Smith R (2018). A peptide-level fully annotated dataset for quantitative evaluation of precursor-aware mass spectrometry data processing algorithms. J Proteome Res.

[11] Gutierrez M, Handy K, Smith R (2018). Quantitative evaluation of algorithms for isotopic envelope extraction via extracted ion chromatogram clustering. J Proteome Res.

[12] Danchilla B. Three.js framework. In: Beginning WebGL for HTML5. New York: Springer; 2012. p. 173–203.

[13] Smith R, Ventura D, Prince JT (2014). Controlling for confounding variables in MS-omics protocol: why modularity matters. Brief Bioinform.

[14] Nahnsen S, Bertsch A, Rahnenführer J, Nordheim A, Kohlbacher O (2011). Probabilistic consensus scoring improves tandem mass spectrometry peptide identification. J Proteome Res.

